# Ellagic Acid Protects Dopamine Neurons via Inhibition of NLRP3 Inflammasome Activation in Microglia

**DOI:** 10.1155/2020/2963540

**Published:** 2020-11-19

**Authors:** Xue-mei He, Yan-zhen Zhou, Shuo Sheng, Jing-jie Li, Guo-qing Wang, Feng Zhang

**Affiliations:** ^1^Key Laboratory of Basic Pharmacology of Ministry of Education and Joint International Research Laboratory of Ethnomedicine of Ministry of Education, Zunyi Medical University, Zunyi, Guizhou, China; ^2^Department of Pharmacology, Key Laboratory of Basic Pharmacology of Guizhou Province and School of Pharmacy, Zunyi Medical University, Zunyi, Guizhou, China; ^3^Department of Ear-Nose-Throat Surgery, The Affiliated Hospital of Zunyi Medical University, Zunyi, Guizhou, China; ^4^Laboratory Animal Center, Zunyi Medical University, Zunyi, Guizhou, China

## Abstract

Neuroinflammation plays a crucial role in the pathological process of Parkinson's disease (PD). Nod-like receptor protein 3 (NLRP3) inflammasome was highly located in microglia and involved in the process of neuroinflammation. Activation of the NLRP3 inflammasome has been confirmed to contribute to the progression of PD. Thus, inhibition of NLRP3 inflammasome activation could be an important breakthrough point on PD therapy. Ellagic acid (EA) is a natural polyphenol that has been widely found in soft fruits, nuts, and other plant tissues with anti-inflammatory, antioxidant, and neuroprotective properties. However, the mechanisms underlying EA-mediated anti-inflammation and neuroprotection have not been fully elucidated. In this study, a lipopolysaccharide- (LPS-) induced rat dopamine (DA) neuronal damage model was performed to determine the effects of EA on the protection of DA neurons. In addition, the DA neuronal MN9D cell line and microglial BV-2 cell line were employed to explore whether EA-mediated neuroprotection was through an NLRP3-dependent mechanism. Results indicated that EA ameliorated LPS-induced DA neuronal loss in the rat substantia nigra. Further, inhibition of microglial NLRP3 inflammasome signaling activation was involved in EA-generated neuroprotection, as evidenced by the following observations. First, EA reduced NLRP3 inflammasome signaling activation in microglia and subsequent proinflammatory cytokines' excretion. Second, EA-mediated antineuroinflammation and further DA neuroprotection from LPS-induced neurotoxicity were not shown upon microglial NLRP3 siRNA treatment. In conclusion, this study demonstrated that EA has a profound effect on protecting DA neurons against LPS-induced neurotoxicity via the suppression of microglial NLRP3 inflammasome activation.

## 1. Introduction

Parkinson's disease (PD) is a chronic progressive neurodegenerative disease characterized by a deep selective loss of dopamine (DA) neurons in the substantia nigra (SN) [1]. Clinical manifestations include static tremor, slow movement, postural instability, stiffness, and other motor disorders [2]. So far, only a few drugs have been available for PD treatment, and most of them just relieve symptoms and could not prevent the death of DA neurons [3].

It was recognized that age-related excessive oxidative stress led to DA autooxidation, *α*-synuclein accumulation, and glial cell activation [4], which are the main causes of neuroinflammation [5]. Further, neuroinflammation, mediated by microglial activation and infiltrating T cells at sites of neuronal injury, was considered to be a prominent contributor to the pathogenesis of progressive PD [6]. Microglia are natural immune barriers in the immune system. While stimulated by external conditions, such as brain damage, inflammation, and pathogens, microglia readily become activated and undergo changes in morphology with hypertrophy and function with phagocytosis. Most importantly, activated microglia could secrete a large number of proinflammatory factors, such as tumor necrosis factor-*α* (TNF-*α*), interleukin-1*β* (IL-1*β*), and IL-18 [7]. The accumulation of these proinflammatory factors contributes to the progressive loss of DA neurons. However, the neurotoxic factors, such as *α*-synuclein, released by the continuing damaged DA neurons, in turn, induce the secondary activation of microglia, and these activated microglia also secrete proinflammatory factors and further cause DA neuronal loss. Thus, a vicious cycle leading to prolonged neuroinflammation and progressive DA neurodegeneration emerges [8]. Collectively, inhibition of microglia-mediated neuroinflammation holds a promising potential for PD treatment.

Recently, inflammasomes are verified as intracellular proinflammatory pattern recognition receptors (PRRs) to initiate and propagate neuroinflammation. As a large multiprotein complex, inflammasome recruits pro-caspase-1 via ASC (the adaptor molecule apoptosis-associated speck-like protein containing a CARD) and then proceeds to cleave the cytokine precursors, such as pro-IL-1*β* and pro-IL-18, into mature IL-1*β* and IL-18 and finally mediates the immune responses against pathogen infection and tissue damage [9]. So far, five different types of inflammasomes have been identified, including NLRP1, NLRP3, NLRC4, Pyrin, and Absent in Melanoma 2 (AIM2) [10]. In particular, the NLRP3 inflammasome has been implicated in the process of neuroinflammation and is highly located in microglia [11]. Increasing evidence revealed that activation of the NLRP3 inflammasome played an important role in the progression of neurodegenerative diseases [12]. Therefore, inhibition of NLRP3 inflammasome activation could be an important breakthrough point in PD therapy.

Ellagic acid (2,3,7,8-tetrahydroxybenzopyrano [5,4,3-cde] benzopyran-5-10-dione, EA) is a polyphenol present in many plants, such as pomegranate plants, grapes, raspberries, blackberries, strawberries, and walnuts. EA exhibits a number of pharmacological activities, such as antioxidant and anti-inflammatory effects [13]. Recent studies confirmed that EA exerted neuroprotection against various neurological disorders. For example, EA generated neuroprotection and improved cognitive dysfunctions in a sporadic Alzheimer's disease (AD) animal model [14]. In addition, EA was indicated to confer neuroprotection against ischemic stroke [15]. However, the underlying mechanisms remain unclear. In this study, rat substantia nigral stereotaxic single injection of lipopolysaccharide- (LPS-) elicited DA neuronal loss was performed to investigate EA-exerted neuroprotection and the underlying mechanisms as well. These findings would provide evidence for the future application of EA on PD treatment.

## 2. Materials and Methods

### 2.1. Reagents

EA (purity > 95%), LPS (Escherichia coli O111:B4), 6-hydroxydopamine (6-OHDA), and apomorphine hydrochloride were obtained from Sigma-Aldrich (St. Louis, CA, USA). Enzyme-linked immunosorbent assay (ELISA) kits for TNF-*α*, IL-1*β*, and IL-18 were bought from Elabscience Biotechnology Co., Ltd. (Wuhan, China). The MTT assay kit was from Beijing Solarbio Science and Technology Co., Ltd. (Beijing, China). Small interfering RNA (siRNA) against NLRP3 was purchased from GenePharma (Shanghai, China). Anti-CR3 complement receptor (OX-42 Catalog No. Ab1211) and tyrosine hydroxylase (TH, Catalog No. Ab113) antibodies were bought from Abcam (Cambridge, MA, USA). Anti-caspase-1 (Catalog No. 22915-1-AP), ionized calcium-binding adapter molecule-1 (Iba-1, Catalog No. 10904-1-AP), *β*-actin (Catalog No. 20536-1-AP), TNF-*α* (Catalog No. 17590-1-AP), IL-1*β* (Catalog No. 66737-1-Ig), IL-18 (Catalog No. 10663-1-AP), rabbit IgG (Catalog No. SA00001-2), and mouse IgG (Catalog No. SA00001-1) antibodies were purchased from the Proteintech Group (Chicago, IL, USA). Anti-NLRP3 (Catalog No. orb101128) antibody was purchased from Biorbyt (Cambridge, United Kingdom).

### 2.2. Animal and Treatment

Male Wistar rats (200–250 g, 8–10 weeks) were bought from the Experimental Animal Center in the Third Military Medical University. All experimental procedures were carried out in accordance with the Chinese Guidelines of Animal Care and Welfare, and this study received approval from the Animal Care and Use Committee of Zunyi Medical University (Zunyi, China). Rats were acclimated to their environment for 1 week before the experiments. All the animals were randomly allocated to five experimental groups with six rats in each group: control, EA alone (50 mg/kg), LPS, LPS+EA (10 mg/kg), and LPS+EA (50 mg/kg). Anesthetized by 7% chloral hydrate (0.5 ml/100 g, *v*/*w*), rats received a single LPS (10 *μ*g in 5 *μ*l PBS) unilateral injection into the SN pars compacta followed by the coordinates 5.2 mm posterior to the bregma, 1.9 mm lateral to the midline, and 8.0 mm ventral to the surface of the skull [16]. EA was intragastrically administrated once a day for 7 consecutive days beginning 30 min before LPS injection. Seven days later, rat behavior changes were analyzed by the rotarod test. Afterwards, animals were sacrificed and the biochemical analysis was performed.

### 2.3. Rotarod Test

Apomorphine-induced rotation was widely employed for assessing the effects of lesions on the dopaminergic system and the success of treatment in PD animal models. Rats were tested for rotational behavior after receiving an intraperitoneal injection of apomorphine hydrochloride (0.5 mg/kg). Subsequently, a rotational behavior test was performed by the cylindrical arrangement containing thin steel rods with two parts by compartmentalization to permit the detection of two rats at the same time. Rats were trained to adapt at a speed of 10 rpm/min before the experiment started, with speed accelerating from 10 to 30 rpm over a period of 5 min until the rat slid off the steps. The duration time each rat stayed on the rod was recorded and calculated for analyzing the behavior changes of rats [17].

### 2.4. Immunohistochemical Analysis and Cell Counting in SN

Rat brains were cut with a horizontal sliding microtome into 35 *μ*m transverse free-floating sections and immunostained with the corresponding antibodies. Then, brain slices were incubated with 0.3% Triton X-100 and blocked with goat serum. Subsequently, brain slices were incubated with anti-TH (1 : 500), OX-42 (1 : 800), and NLRP3 (1 : 800) antibodies at 4°C overnight, respectively [18]. Digital images of TH-positive neurons, OX-42-positive microglia, and NLRP3-positive inflammasomes in midbrain SN were acquired by an Olympus microscope (Olympus, Tokyo, Japan). Representative fluorescence images were obtained, and the fluorescence intensity was calculated using ipwin32 software.

### 2.5. Cell Culture and Treatment

The mouse microglial BV-2 cell line was obtained from the China Center for Type Culture Collection (Wuhan, China). Cultures were maintained in minimum essential medium (MEM) supplemented with 10% heat-inactivated fetal bovine serum (FBS), 100 U/ml penicillin, and 100 *μ*g/ml streptomycin at 37°C in the humidified atmosphere of 5% CO_2_ and 95% air [19]. The DA neuronal MN9D cell line was purchased from the Cell Culture Center in the Institute of Basic Medical Sciences of the Chinese Academy of Medical Sciences (Beijing, China). MN9D cells were cultured in DMEM with 10% FBS and 1% penicillin-streptomycin on an atmosphere with 5% CO_2_ at 37°C in the humidified atmosphere of 5% CO_2_ and 95% air [20].

### 2.6. MTT Assay

Cell viability was evaluated by MTT assay. BV-2 and MN9D cells were cultured in 1 × 10^5^/well in 96-well plates for 24 h. Afterwards, cells were treated with different concentrations of EA for 30 min followed by LPS (100 ng/ml) or 6-OHDA (100 *μ*M) treatment for 24 h and then incubated with MTT solution (5 g/l) for 4 h. Formazan crystals in the cells were solubilized using 200 *μ*l dimethyl sulfoxide (DMSO), and the absorbance was detected by an automated microplate reader within a 490 nm wavelength [21].

### 2.7. Western Blot Analysis

Total protein content was extracted from rat midbrain tissue and BV-2 cells using a lysis buffer containing protease inhibitors. Protein levels were quantified by BCA [22]. Protein (10 *μ*g) from each sample was subjected to SDS-PAGE gel under the reduced conditions. Proteins were then transferred onto polyvinylidene fluoride (PVDF) membranes. The membranes were blocked with 5% nonfat milk for 2 h at room temperature and then incubated overnight at 4°C with the following primary antibodies: Iba-1 (1 : 800), NLRP3 (1 : 800), caspase-1 (1 : 800), TNF-*α* (1 : 1000), IL-1*β* (1 : 500), IL-18 (1 : 800), and *β*-actin (1 : 2000). Next, the membranes were incubated for 1 h with a horseradish peroxidase-conjugated anti-mouse IgG antibody or anti-rabbit IgG at 1 : 2000 dilution. The blot films were developed with an enhanced ECL Reagent.

### 2.8. ELISA

The levels of TNF-*α*, IL-1*β*, and IL-18 were measured by ELISA according to the manufacturer's instructions. The microplate reader was used to measure the absorbance at 450 nm.

### 2.9. Immunofluorescence Staining

Activated microglia were identified with an anti-OX-42 antibody. Cells were fixed with paraformaldehyde (4%) for 30 min. Later, cells were permeabilized using Triton X-100 (0.3%) for 15 min. Then, cells were blocked using a goat serum blocking solution for 60 min at 37°C. Thereafter, cells were incubated with 1 : 800 dilution of anti-OX-42 antibody overnight at 4°C. Following overnight incubation, cells were incubated in the dark for 30 min with goat anti-rabbit secondary antibody (1 : 1000) or goat anti-mouse secondary antibody (1 : 1000). Cells were also counterstained with DAPI for 5 min [23]. After rinsing cells with PBS, representative fluorescence images were obtained using an EVOS® Floid® Cell Imaging Station. The fluorescence intensity was calculated by using ipwin32 software.

### 2.10. RNA Transfection

BV-2 cells were cultured and seeded in a 6-well plate at a density of 1 × 10^5^ cells/ml. The transfection of siRNA was performed complying with the manufacturer's protocol. A total of 2 *μ*l of NLRP3 siRNA was diluted into 18 *μ*l of transfection medium. GP-siRNA-Mate plus (180 *μ*l) was used to transfect with siRNA dilution (20 *μ*l). After transfection for 6 h, the transfection solution was removed and cells were rinsed with PBS and replaced with MEM containing 2% FBS and then treated with EA for 30 min followed by LPS (100 ng/ml) treatment for 24 h [19]. The gene sequences were as follows: sense, 5′-UUC UCC GAA CGU GUC ACG UTT-3′; antisense, 5′-ACG UGA CAC GUU CGG AGA ATT-3′.

### 2.11. Statistical Analysis

Results were indicated as the mean ± standarderrorofthemean(SEM). Statistical significance was analyzed by one-way analysis of variance (ANOVA) using GraphPad Prism software (GraphPad Software Inc., San Diego, CA, USA). Upon ANOVA demonstrating the significant differences, pairwise comparison between means was evaluated by Bonferroni's *post hoc* test with correction. A value of *p* < 0.05 was considered statistically significant.

## 3. Results

### 3.1. EA Attenuated LPS-Induced DA Neuronal Damage in SN *In Vivo*

**Figure 1:**
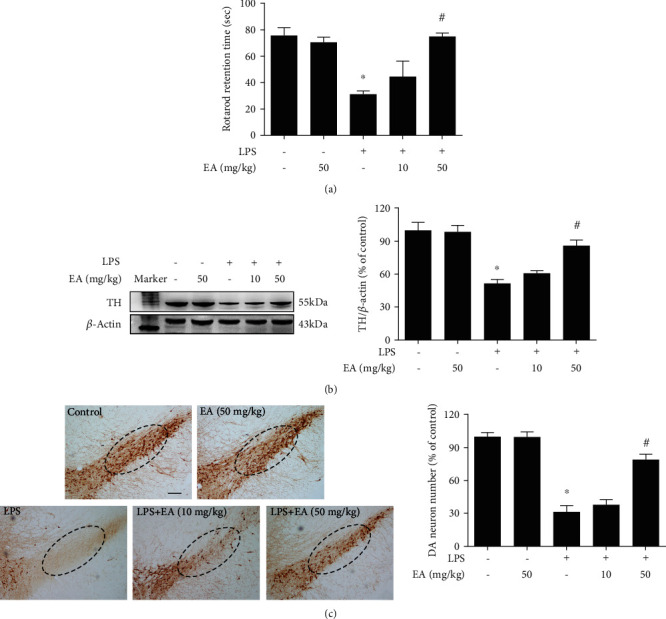
EA attenuated LPS-induced DA neuronal damage in SN *in vivo*. Rats were intragastrically given EA (50 mg/kg) for 7 consecutive days. Rat behavior changes were analyzed by the rotarod test (a). TH protein expression in the rat midbrain was tested by western blot assay (b). Brain sections were immunostained with an anti-TH antibody, and the number of TH-positive neurons in SN was counted (c). The “ellipse” presented the area of SN. Scalebar = 200*μ*m. Data were the mean ± SEM from 6 rats. ^∗^*p* < 0.05 compared with the control group; ^#^*p* < 0.05 compared with the LPS group.

Neuroprotective effects of EA on LPS-induced DA neuronal damage were investigated in rats. As shown in Figure 1(a), LPS reduced the time rats stayed on the rod, compared with the control group. However, EA (50 mg/kg) attenuated the LPS-caused decrease in the time rats remained on the rod, while no significant protection of EA (10 mg/kg) was discerned. To further confirm EA-mediated DA neuroprotection, the TH-positive neuronal number and TH protein expression were determined. As shown in Figure 1(b), EA (50 mg/kg) ameliorated the LPS-induced decrease in TH protein expression. Consistent with TH protein detection results, TH-positive neuronal counting indicated that EA exerted neuroprotection against LPS-induced DA neuronal loss (Figure 1(c)).

### 3.2. EA Ameliorated LPS-Elicited Activation of Microglia and NLRP3 Inflammasome Signaling *In Vivo*

**Figure 2:**
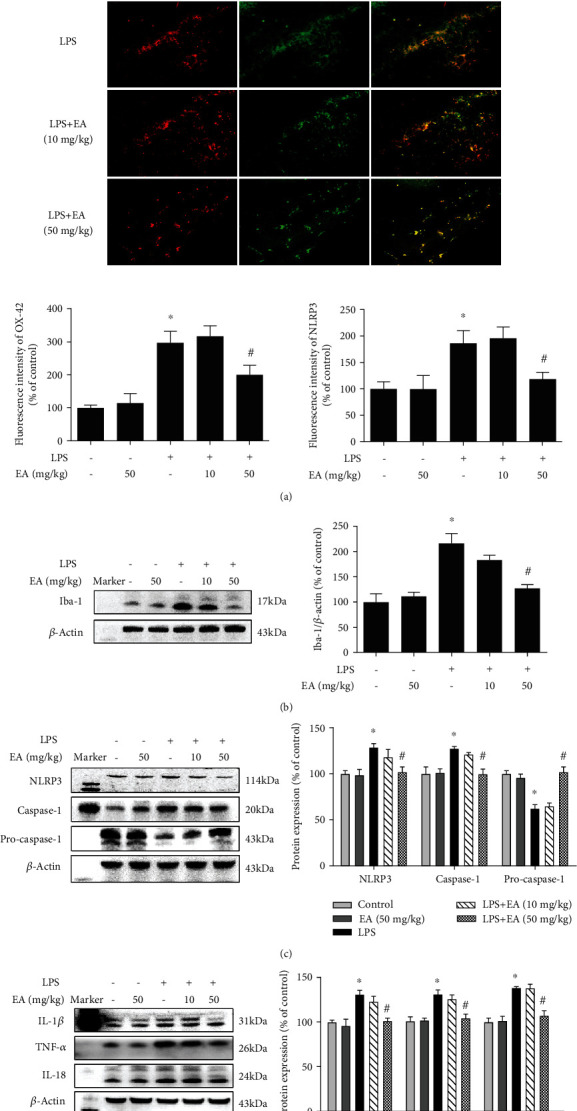
EA ameliorated LPS-elicited activation of microglia and NLRP3 inflammasome signaling *in vivo*. Rat brains were collected and stained by double immunofluorescence with anti-NLRP3 and anti-OX-42 antibodies (green fluorescence represented NLRP3 inflammasome, and red fluorescence represented microglia) (a). The protein expressions of Iba-1 (b); NLRP3, caspase-1, and pro-caspase-1 (c); and TNF-*α*, IL-1*β*, and IL-18 (d) in the rat midbrain were determined via western blot assay. Data were the mean ± SEM from 6 rats and expressed as a percentage of the control group. ^∗^*p* < 0.05 compared with the control group; ^#^*p* < 0.05 compared with the LPS group.

Next, the effects of EA on microglia and NLRP3 inflammasome activation were investigated. To verify the connection between the NLRP3 inflammasome and microglia, the double-immunofluorescence calibration site was conducted. As shown in Figure 2(a), the NLRP3 inflammasome was activated and located in activated microglia. EA attenuated LPS-induced activation of the NLRP3 inflammasome in microglia. Also, EA inhibited Iba-1 protein expression induced by LPS (Figure 2(b)). In addition, EA suppressed LPS-induced activation of NLRP3 inflammasome signaling (Figure 2(c)) and proinflammatory cytokine (IL-1*β*, TNF-*α*, and IL-18) protein expressions (Figure 2(d)).

### 3.3. EA Had No Direct Neuroprotective Effects on DA Neurons

**Figure 3:**
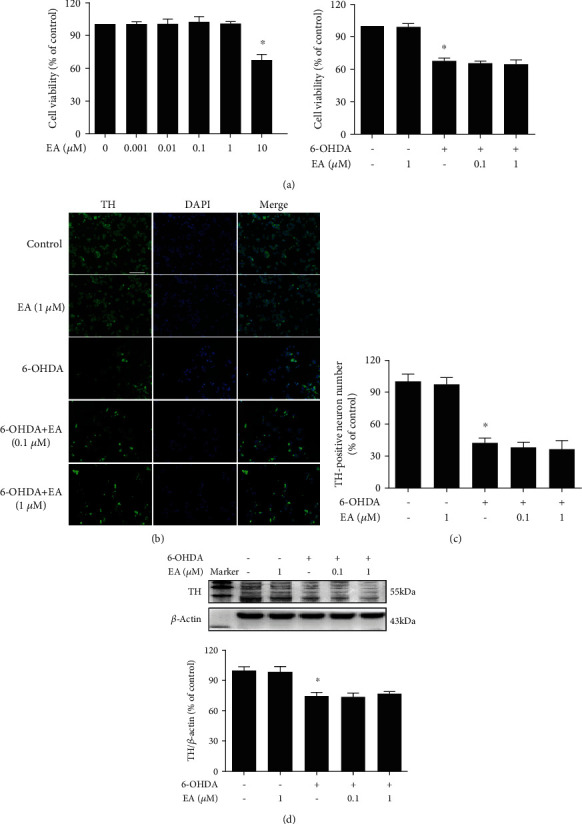
EA had no direct neuroprotective effects on DA neurons. MN9D cells were treated with EA (0.1 and 1 *μ*M) for 30 min and then incubated with 6-OHDA (100 *μ*M) for 24 h. Cell viability was determined by MTT assay (a). 6-OHDA-induced MN9D cell damage was evaluated by immunostaining (b) and cell counting (c). Scalebar = 100*μ*m. The protein expression of TH was detected by western blot assay (c). Data were the mean ± SEM from three independent experiments performed in triplicate. ^∗^*p* < 0.05 compared with control cultures; ^#^*p* < 0.05 compared with 6-OHDA-treated cultures.

To further confirm whether EA produced direct neuroprotective actions on DA neurons, the effects of EA on 6-OHDA-induced DA neuronal damage *in vitro* were determined. First, as shown in Figure 3(a), 6-OHDA-induced neurotoxicity was not attenuated by EA treatment in MN9D cell-enriched cultures. Next, in TH-positive neuronal counting analysis (Figures 3(b) and 3(c)), 6-OHDA caused TH-positive neuronal loss and EA did not confer protection from 6-OHDA-induced neuronal damage. Similar results were indicated in the TH protein detection shown in Figure 3(d). These results demonstrated that EA did not generate direct neuroprotection on DA neurons.

### 3.4. EA Inhibited Microglial NLRP3 Inflammasome Activation *In Vitro*

**Figure 4:**
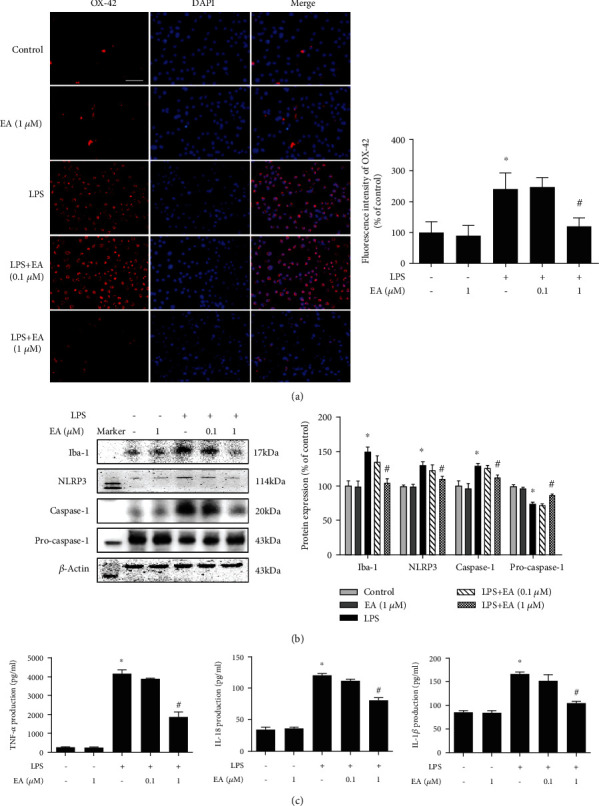
EA inhibited microglial NLRP3 inflammasome activation *in vitro*. BV-2 cells were treated with EA (0.1 and 1 *μ*M) for 30 min and then incubated with LPS (100 ng/ml) for 24 h. Microglial activation was evaluated by immunostaining with an anti-OX-42 antibody (a) and quantitated by western blot analysis with an anti-Iba-1 antibody (b). Scalebar = 100*μ*m. The effects of EA on NLRP3 inflammasome signaling activation in BV-2 cells were detected via western blotting (b). The ratios of densitometry values of Iba-1, NLRP3, caspase-1, and pro-caspase-1 with *β*-actin were analyzed and normalized to each respective control cultures. The release of proinflammatory factors, such as TNF-*α*, IL-1*β*, and IL-18, in BV-2 cell culture medium was measured by ELISA (c). Data were the mean ± SEM from three independent experiments performed in triplicate. ^∗^*p* < 0.05 compared with control cultures; ^#^*p* < 0.05 compared with LPS-treated cultures.

The effects of EA on microglia and NLRP3 inflammasome signaling activation were further confirmed *in vitro*. First, BV-2 cells were employed to examine the effects of EA on LPS-induced microglial activation. As shown in Figure 4(a), immunofluorescence staining assay indicated that EA reduced LPS-induced microglial activation. Also, EA decreased LPS-induced higher protein expression of Iba-1 (Figure 4(b)). Then, EA inhibited the activation of microglial NLRP3 inflammasome signaling induced by LPS (Figure 4(b)). In addition, EA eliminated LPS-induced production of proinflammatory factors, such as TNF-*α*, IL-1*β*, and IL-18, in the culture medium (Figure 4(c)).

### 3.5. NLRP3 Inflammasome Signaling Inactivation Was Involved in EA-Mediated Anti-Inflammatory Properties

**Figure 5:**
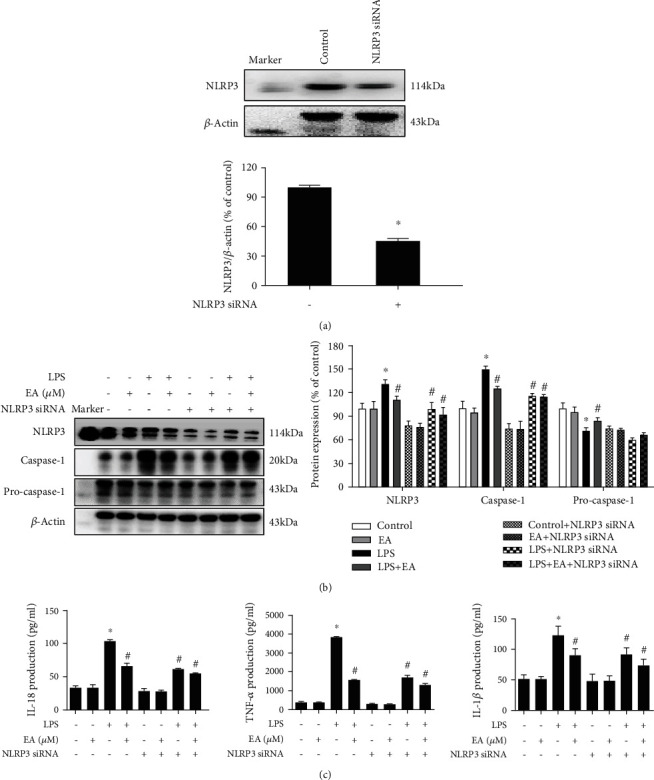
NLRP3 inflammasome signaling inactivation was involved in EA-mediated anti-inflammatory properties. BV-2 cells were treated with NLRP3 siRNA (40 nM). After 6 h of transfection, the transfection solution was removed and cells were rinsed with PBS. The silencing efficiency was assessed via NLRP3 protein expression detection (a). Moreover, BV-2 cells were treated with EA (1 *μ*M) in the presence of NLRP3 siRNA and then exposed to LPS for 24 h. The protein expressions of Iba-1, NLRP3, caspase-1, and pro-caspase-1 in BV-2 cells were detected via western blot assay (b). The levels of TNF-*α*, IL-1*β*, and IL-18 in the culture medium were measured by ELISA (c). Data were the mean ± SEM from three independent experiments performed in triplicate. ^∗^*p* < 0.05 compared with control cultures; ^#^*p* < 0.05 compared with LPS-treated cultures.

To investigate the role of NLRP3 inflammasome signaling in EA-mediated antineuroinflammation, NLRP3 siRNA was performed in BV-2 cell cultures. First, as shown in Figure 5(a), NLRP3 siRNA was transfected into BV-2 cells and the successful transfection with NLRP3 siRNA was evaluated by the NLRP3 protein level. After NLRP3 siRNA administration, both NLRP3 siRNA and EA inhibited LPS-induced NLRP3 inflammasome signaling activation. However, no significant difference of NLRP3 and caspase-1 protein expressions between the LPS+EA and LPS+EA+NLRP3 siRNA groups was discerned (Figure 5(b)). In addition, the effects of EA on proinflammatory factors' excretion with NLRP3 siRNA application were measured. In parallel with NLRP3 inflammasome signaling analysis, EA did not reduce LPS-induced release of proinflammatory factors again after NLRP3 siRNA treatment (Figure 5(c)). These observations indicated EA attenuated microglia-induced neuroinflammation via inhibition of NLRP3 inflammasome signaling activation.

### 3.6. EA Targeted Microglial NLRP3 Inflammasome to Produce DA Neuroprotection

**Figure 6:**
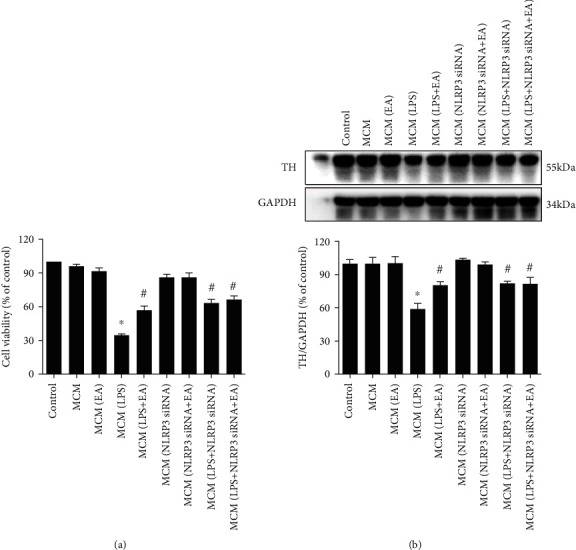
EA targeted microglial NLRP3 inflammasome to produce DA neuroprotection. Microglia-conditioned medium (MCM) prepared from BV-2 cell cultures with administration of EA (MCM (EA)), LPS (MCM (LPS)), LPS+EA (MCM (LPS+EA)), NLRP3 siRNA (MCM (NLRP3 siRNA)), NLRP3 siRNA+EA (MCM (NLRP3 siRNA+EA)), NLRP3 siRNA+LPS (MCM (NLRP3 siRNA+LPS)), and LPS+NLRP3 siRNA+EA (MCM (LPS+NLRP3 siRNA+EA)) was harvested and added to MN9D cells incubated for 24 h. MN9D cell viability was determined by MTT assay (a). TH protein expression was tested by western blot assay (b). Data were the mean ± SEM from three independent experiments performed in triplicate. ^∗^*p* < 0.05 compared with control cultures; ^#^*p* < 0.05 compared with MCM (LPS)-treated cultures.

Since microglia were the target of EA-generated DA neuroprotection, whether this neuroprotection resulted from inhibiting microglial NLRP3 inflammasome activation was then explored. As shown in Figure 6, compared with the MCM (LPS) group, both MCM (LPS+NLRP3 siRNA) and MCM (LPS+EA) protected against MCM (LPS)-induced neurotoxicity evidenced by cell viability and TH protein expression detection, whereas no significant difference of neuroprotection between these two groups was exhibited. Further, MCM (LPS+NLRP3 siRNA+EA) did not exert more DA neuroprotection against MCM (LPS)-caused neuronal injury than MCM (LPS+NLRP3 siRNA) or MCM (LPS+EA) treatment.

## 4. Discussion

**Figure 7:**
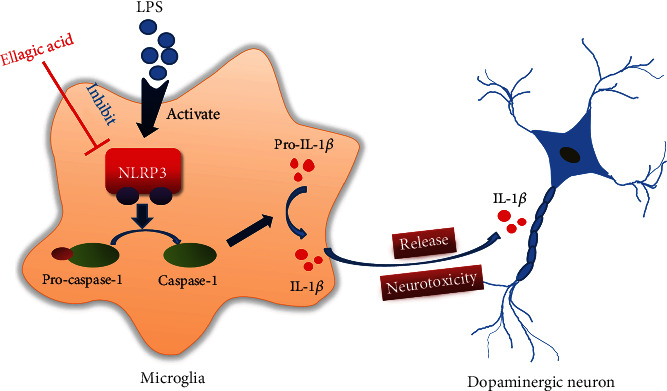
The mechanisms underlying EA-mediated dopamine neuroprotection.

The present study is aimed at investigating the neuroprotective actions of EA on LPS-induced DA neuronal loss and evaluating the role of microglia-mediated neuroinflammation in this neuroprotection. Results indicated that EA protected DA neurons against LPS-induced neurotoxicity in SN. Further, inhibition of microglial NLRP3 inflammasome signaling activation was involved in EA-generated neuroprotection, as evidenced by the following observations. First, EA reduced NLRP3 inflammasome signaling activation in microglia and subsequent proinflammatory cytokines' excretion. Second, EA-mediated antineuroinflammation and further DA neuroprotection from LPS-induced neurotoxicity were not shown upon microglial NLRP3 siRNA treatment. Taken together, EA conferred neuroprotection against LPS-induced DA neuronal damage via inhibition of microglial NLRP3 inflammasome signaling activation (Figure 7).

Neuroinflammation is considered to be the most common feature of the aging brain and neurodegenerative disorders, including AD, PD, and amyotrophic lateral sclerosis (ALS) [5]. It is primarily mediated by activated glial cells, especially for microglia, and accompanied by the secretion of proinflammatory mediators [24]. As the first defense of immune surveillance, microglia readily become activated and predominately participate in the inflammatory response. In addition, astroglia could amplify microglia-mediated neuroinflammation and result in the feedback loop of neuroinflammatory reactions [25]. In this regard, understanding the molecular mechanisms of PD can be found by investigating microglia-mediated neuroinflammation [26]. Therefore, to target microglia-derived proinflammatory cytokines might offer promising therapeutic approaches for PD management. In this study, EA protected DA neurons against LPS-induced neurotoxicity and reduced microglial proinflammatory factors' release, suggesting that EA-generated DA neuroprotection was closely associated with the inhibition of microglia-mediated neuroinflammation. Consistent with this finding, EA was previously confirmed to reduce the production of the proinflammatory cytokines, such as TNF-*α* and IL-6, in LPS-stimulated RAW 264.7 macrophage cells [27]. Furthermore, the dose of EA (50 mg/kg) presented beneficial effects on protection against LPS-induced DA neurotoxicity and inhibition of microglial NLRP3 inflammasome activation, whereas significant effects mediated by EA (10 mg/kg) were shown. This dose profile suggested that EA (10-50 mg/kg) was under the effective dose range in rats and guided that the selection of doses of EA complied with the dose-effect relationship. However, additional clinical studies are indeed needed to validate the dose range and dose-effect relationship of EA on humans corresponding to that in rats.

In addition, inflammasomes are multiprotein complexes responsible for intracellular sensors of environmental and cellular stress [28, 29]. As the inflammasome family member, the NLRP3 inflammasome is mainly involved in the response of neuroinflammation [11]. Upon cellular stress, assembly of NLRP3 inflammasome triggers caspase-1 activation and further caspase-1-mediated production of IL-1*β* and IL-18, thereby initiating neuroinflammation [30]. In PD patient brains, the NLRP3 inflammasome was potentially activated by insoluble *α*-synuclein aggregates and oxidative stress [31]. However, deficiency of NLRP3 inflammasome attenuated motor dysfunction and DA neurodegeneration in a PD mouse model [32]. Thus, inhibition of NLRP3 inflammasome activation might be beneficial for PD intervention. The present study demonstrated that EA could inhibit NLRP3 inflammasome signaling activation. Similar results exhibited that EA prevented monocrotaline-induced rat pulmonary artery hypertension via inhibiting the activation of the NLRP3 inflammasome and caspase-1 in the lungs and release of proinflammatory cytokines, such as IL-*β*, in serum [33]. On the other hand, this study indicated that EA-mediated DA neuroprotection was present in neuron-microglia cocultures but not in neuron-enriched cultures, implying that microglia were at least essential for EA-generated neuroprotection. Moreover, EA was found to reduce LPS-induced proinflammatory factors' production. Collectively, EA-inhibited microglial activation, and the subsequent proinflammatory factors' release might be attributed to the inhibition of microglial NLRP3 inflammasome activation. This conclusion was verified by the following observations: (1) EA inhibited microglial NLRP3 and pro-caspase-1 activation and IL-1*β* production; (2) EA could not further suppress LPS-induced proinflammatory factors' release and produce DA neuroprotection after neuron-microglia cocultures treated by NLRP3 siRNA.

Interestingly, EA not only decreased IL-*β* and IL-18 production via inhibition of NLRP3 inflammasome activation but also reduced TNF-*α* secretion. Why did EA suppress TNF-*α* production during EA-inhibited NLRP3 inflammasome activation? The amount of evidence indicated that the NLRP3 inflammasome participated in NF-*κ*B-mediated inflammatory processes of diseases [34, 35], which suggested that there was crosstalk between the NLRP3 inflammasome and NF-*κ*B signaling pathways. Thus, we speculated that EA-reduced TNF-*α* production might be associated with the regulation of the NLRP3 inflammasome/NF-*κ*B signaling.

To date, current PD therapy is focused on symptom control and fails to delay the progressive neurodegenerative process. Actually, various side effects of the available drugs present huge challenges for long-term application. Therefore, more potential therapeutic candidates are urgently essential for halting the progression of PD. Recent studies demonstrated that inhibition of neuroinflammation would attenuate DA neurodegeneration. Thus, anti-inflammatory agents might provide new avenues for PD treatment. However, the low success of translating promising anti-inflammatory candidates from animal studies to clinical trials was indicated. Therefore, an urgent approach for a novel anti-inflammatory alternative design was prompted [28]. Since activation of the microglial NLRP3 inflammasome was verified to play a pivotal role in the progression of neurodegenerative disorders, such as AD, PD, and ALS, inhibition of NLRP3 inflammasome activation might become a promising therapeutic target for these neurodegenerative disorders. Here, the present study demonstrated that EA conferred DA neuroprotection against LPS-induced neurotoxicity and modulation of microglial NLRP3 inflammasome signaling activation was revealed to participate in this neuroprotection. These findings suggested that EA could open a new window on neurodegenerative disorder treatment. However, the current study on EA only stays in animal experiments, and the follow-up hopes to be used as a clinical drug application for future studies.

## 5. Conclusion

This study demonstrated that EA has a profound effect on protecting DA neurons against LPS-induced neurotoxicity via suppression of microglial NLRP3 inflammasome signaling activation. These findings suggest that EA might be a potential benefit for PD treatment.

## Data Availability

Data in this manuscript were available from the corresponding author on reasonable request.

## Abbreviations

6-OHDA: 6-Hydroxydopamine
AD: Alzheimer's disease
DA: Dopamine
DMSO: Dimethyl sulfoxide
EA: Ellagic acid
ELISA: Enzyme-linked immunosorbent assay
FBS: Fetal bovine serum
Iba-1: Ionized calcium-binding adapter molecule-1
IL-1*β*: Interleukin-1*β*
IL-18: Interleukin-18
LPS: Lipopolysaccharide
MCM: Microglia-conditioned medium
MEM: Minimum essential medium
NLRP3: Nod-like receptor protein 3
OX-42: Anti-CR3 complement receptor
PBS: Phosphate-buffered saline
PD: Parkinson's disease
PVDF: Polyvinylidene fluoride
siRNA: Small interfering RNA
SN: Substantia nigra
TH: Tyrosine hydroxylase
TNF-*α*: Tumor necrosis factor-*α*.
